# Muscle Fiber Type-Predominant Promoter Activity in Lentiviral-Mediated Transgenic Mouse

**DOI:** 10.1371/journal.pone.0016908

**Published:** 2011-03-18

**Authors:** Tomohiro Suga, En Kimura, Yuka Morioka, Masahito Ikawa, Sheng Li, Katsuhisa Uchino, Yuji Uchida, Satoshi Yamashita, Yasushi Maeda, Jeffrey S. Chamberlain, Makoto Uchino

**Affiliations:** 1 Department of Neurology, Graduate School of Medical Sciences, Kumamoto University, Kumamoto, Japan; 2 Research Center for Infection-Associated Cancer, Division of Disease Model Innovation, Institute for Genetic Medicine, Hokkaido University, Sapporo, Japan; 3 Research Institute for Microbial Diseases, Osaka University, Osaka, Japan; 4 Department of Neurology, Medicine, and Biochemistry, University of Washington School of Medicine, Seattle, Washington, United States of America; 5 Laboratory of Pharmacology, Division of Life Science, Faculty of Pharmaceutical Sciences, Sojo University, Kumamoto, Japan; Instituto de Ciencia de Materiales de Madrid - Instituto de Biomedicina de Valencia, Spain

## Abstract

Variations in gene promoter/enhancer activity in different muscle fiber types after gene transduction was noticed previously, but poorly analyzed. The murine stem cell virus (MSCV) promoter drives strong, stable gene expression in hematopoietic stem cells and several other cells, including cerebellar Purkinje cells, but it has not been studied in muscle. We injected a lentiviral vector carrying an MSCV-EGFP cassette (LvMSCV-EGFP) into tibialis anterior muscles and observed strong EGFP expression in muscle fibers, primary cultured myoblasts, and myotubes isolated from injected muscles. We also generated lentiviral-mediated transgenic mice carrying the MSCV-EGFP cassette and detected transgene expression in striated muscles. LvMSCV-EGFP transgenic mice showed fiber type-dependent variations in expression: highest in types I and IIA, intermediate in type IID/X, and lowest in type IIB fibers. The soleus and diaphragm muscles, consisting mainly of types I and IIA, are most severely affected in the *mdx* mouse model of muscular dystrophy. Further analysis of this promoter may have the potential to achieve certain gene expression in severely affected muscles of *mdx* mice. The Lv-mediated transgenic mouse may prove a useful tool for assessing the enhancer/promoter activities of a variety of different regulatory cassettes.

## Introduction

A recombinant lentiviral vector is an efficient tool for delivering genes to dividing and non-dividing cells [1], [2], [3]. Compared with non-integrating vectors, the ability of lentiviral vectors to integrate is an important advantage when targeting muscle diseases, such as Duchenne muscular dystrophy (DMD) [4], [5], [6], [7], [8], [9], [10]. DMD muscles lack the dystrophin protein, and dystrophin gene replacement is expected to halt ongoing myofiber turnover. To drive the transcription of various genes in gene therapy protocols, several strong viral enhancer/promoters were used in previous studies, including cytomegalovirus (CMV) [11], [12],[13], Raus sarcoma virus (RSV) [14], [15], [16], cytomegalovirus enhancer, chicken beta-actin (CAG) promoter [16], [17], [18], [19], murine stem cell virus (MSCV) [16], [17], [20], [21], mouse phosphoglycerate-kinase 1 (pGK) [12], [22], [23], and elongation factor 1 (EF1) [12], [24], which are ubiquitous promoters derived from house keeping gene regulation systems in mammalian cells. As the MSCV promoter drives gene expression as strongly as the CMV promoter and achieves stable gene expression in different type of cells, especially muscle fibers, we previously used the MSCV promoter to regulate the expression of a short version of the dystrophin gene in a lentiviral vector [6]. The expression of therapeutic protein was detected in *mdx* muscles, however it was a little weaker in some of the fibers than we expected. Similarly, when we intramuscularly injected a lentiviral vector containing an MSCV-EGFP cassette into murine skeletal muscles, we observed a variety of EGFP expression levels [6]. The MSCV promoter has also been used in the hematopoietic gene therapy field [17], [20], and was reported to be the best for viral-vector-mediated gene delivery into cerebellar Purkinje cells *in vivo* [16]; nevertheless, no other assessment of its tissue specificity was previously reported.

As the muscle creatine kinase gene (*CK6*) promoter, a muscle-specific promoter [25], has fiber-type-dependent activity in murine skeletal muscle when tested in AAV vectors [26], [27], we hypothesized that the observed weaknesses in MSCV promoter activity might also depend on muscle fiber type. Murine skeletal muscles are composed of four major fiber types: type I or slow-twitch oxidative, IIA or fast-twitch oxidative glycolytic, IID/X or fast-twitch intermediate glycolytic, and IIB or fast-twitch glycolytic fibers. The fiber types can be distinguished by myosin-ATPase, oxidative enzyme histochemistry, and nicotinamide adenine dinucleotide tetrazolium reductase (NADH-TR) reactions [28], and are related to two distinct physiological parameters: speed of contraction and resistance to fatigue [29], [30].

Muscle fiber types with predominant promoter activity for vector-mediated gene therapy have been noted but not yet extensively studied. As we are interested in the usefulness of the strong MSCV promoter to drive therapeutic genes, such as *dystrophin*, in skeletal muscles, we have focused on the activity of the MSCV promoter in skeletal muscles and cultured myotubes. To explore the gene regulating ability of the MSCV promoter in skeletal muscles, we tested lentiviral gene delivery methods with intramuscular injection. Ikawa et al. previously reported that lentiviral-mediated transgenic mice were easy to generate and might be a useful tool for analyzing gene expression cassettes, including enhancer/promoter gene regulation abilities [31]. Therefore, we also generated a transgenic mouse carrying the MSCV-EGFP cassette using lentiviral-mediated gene delivery to two-cell-stage embryos and performed detailed analyses of this mouse, including the pattern of EGFP expression in skeletal muscles.

## Results

### Titer of viral vectors

**Figure 1:**
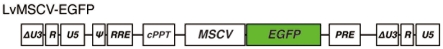
Construction of a lentiviral vector. *EGFP* was packaged into the lentiviral vector under the control of an MSCV promoter. The titer of the virus was estimated at about 1.0×10^9^ TU/ml. Ψ; packaging signal, RRE; HIV Rev response element, cPPT; HIV-1 central polypurine tract, MSCV; murine stem cell virus promoter, EGFP; enhanced green fluorescence protein gene, PRE; human hepatitis virus post-transcriptional regulatory element.

Before using the lentiviral vectors in *in vivo* and *in vitro* experiments, we analyzed their ability to transduce 293D and NIH3T3 cells [6]. The titer of the virus was estimated to be 5.0×10^8^−2.0×10^9^ transduction units (TU)/ml in a series of preparations (Figure 1, legend).

### Variations in EGFP expression levels after intramuscular injection of LvMSCV-EGFP

**Figure 2:**
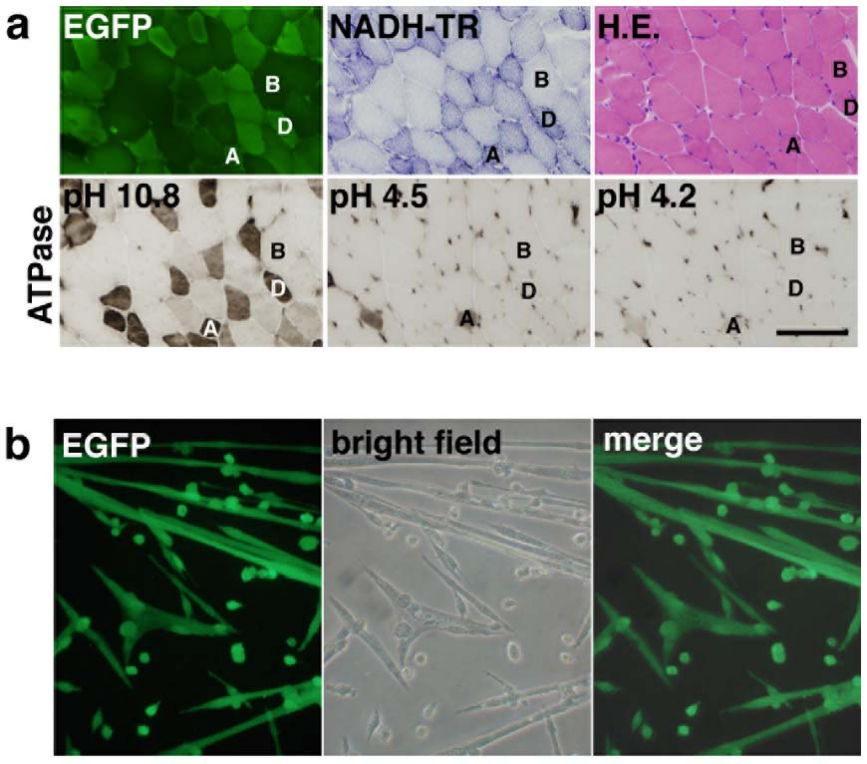
Intramuscular injection of LvMSCV-EGFP into striated muscle. (**a**) Micrographs of serial sections from a lentiviral vector-transduced skeletal muscle showing fluorescent EGFP expression and NADH-TR, HE, and ATPase staining (at pHs 10.8, 4.5, and 4.2). EGFP expression was strong in the Type IID/X fibers (D), which can be distinguished by dark staining with NADH-TR and ATPase at pH 10.8, but weaker in type IIB fibers (B), which are lightly stained in NADH-TR. There are a few type IIA fibers (A), darkly stained with NADH-TR and ATPase at pHs 10.8, 4.5, and 4.2, also brightly positive for EGFP. Scale bar represents 100 µm. (**b**) Primary cultured myotubes isolated from LvMSCV-EGFP-injected TA muscles. Seven weeks after transduction, muscle cells were isolated and cultured in F10C/15% HS/5 ng/ml with bFGF. EGFP-positive myotubes were observed after 7 days in culture.

To determine whether the lentiviral vectors could efficiently mediate transgene expression in mouse skeletal muscles under the control of the MSCV promoter, we injected 5 µl of LvMSCV-EGFP vector into the TA muscles of 3-day-old C57Bl/10 mice. By 4 weeks post injection, EGFP expression was observed in the injected muscles. Although there were differences in the overall expression levels in individual muscles, among the independent injections, a differential expression could still be observed in the various muscle fiber types. In particular, the type IID/X fibers, which could be distinguished as darkly stained fibers with NADH-TR and ATPase (pH 10.8) staining, expressed higher levels of EGFP than did the type IIB fibers, which were lightly stained with NADH-TR (Figure 2a).

### EGFP expression in primary myoblasts and myotubes cultured from the injected muscles

To test whether intramuscularly injected lentiviral vector reached the myogenic progenitor cells in skeletal muscles, we harvested injected muscles, digested them with collagenase, and collected mononuclear cells to culture in dishes. Cells proliferated well and then formed myotubes. About 10–40% of the myoblast colonies were positive for EGFP. In the positive colonies, cells continued this expression during the time that they fused and formed myotubes (Figure 2b). Thus, primary cultured myoblasts and myotubes highly expressed EGFP driven by the MSCV promoter.

### Efficiency and variation of EGFP expression in mice derived from Lv-transduced embryos

To analyze MSCV promoter activity without the influence of the variability we observed among the individual manual injections, we transduced the lentiviral vectors into B6D2F1 mouse embryos and generated 8 transgenic mouse lines that included lentiviral vector provirus genomes in their chromosomes. From two-cell-stage embryos transduced with LvMSCV-EGFP, 17 F0 mice were born, of which 8 were positive for the for EGFP transgene (genomic PCR analysis, Table S1). The mice were sacrificed at 8–10 mo, and then analyzed by quantitative PCR to determine the numbers of Lv-provirus copies inserted in the host cell genomes. In this case, a mean of 2.70±0.75 Lv-provirus copies were stably integrated into the mouse genomes.

### Type I and type IIA fibers contain the predominant EGFP expression in LvMSCV-EGFP transgenic mouse lines

**Figure 3:**
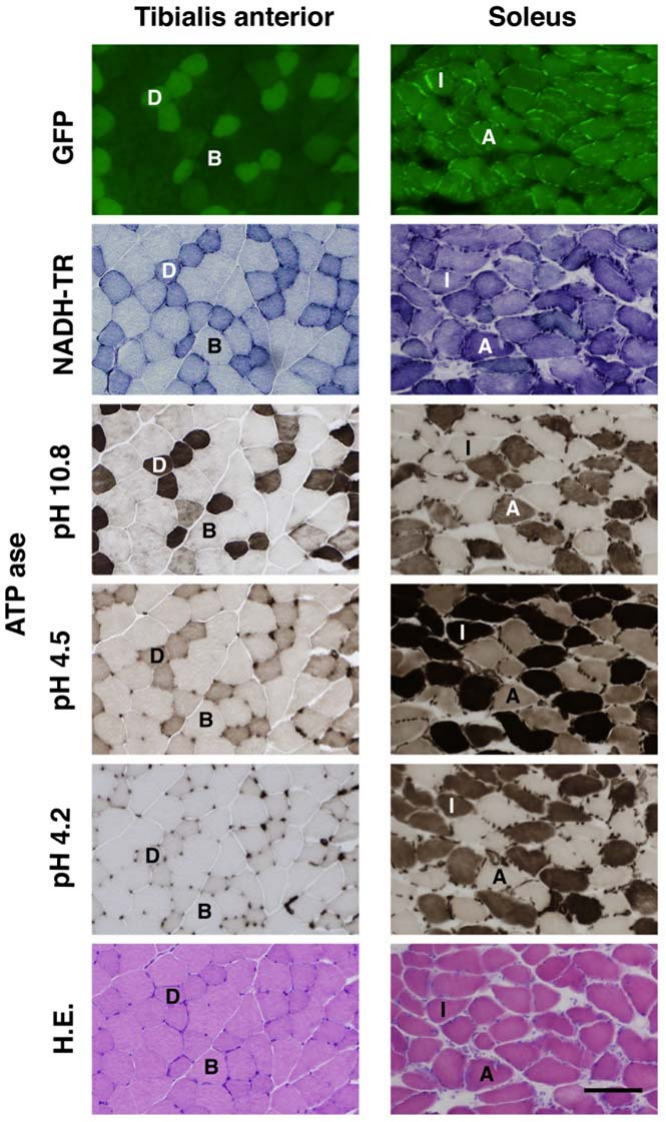
Type I, IIA, and IID/X fiber-predominant EGFP expression in the tibialis anterior and soleus muscles from lentiviral-mediated MSCV-EGFP transgenic mouse. Micrographs from serial-sectioned TA and soleus muscles from 3-month-old LvMSCV-EGFP transgenic mice showing EGFP fluorescence, NADH-TR, ATPase (at pHs 10.8, 4.5, and 4.2), and HE staining. In the TA muscle (*left*) dappled EGFP expression is observed; EGFP expression in strongest in type IID/X fibers (labeled D) and weaker in type IIB fibers (labeled B). The TA muscle consists of mainly type IIB and IID/X fibers. On the other hand, soleus muscles (*right*) consist mainly of type I and IIA fibers (labeled I and A, respectively), both of which strongly express EGFP. The fiber types were confirmed by NADH-TR, and ATPase (pH 10.8, 4.5 and 4.2) staining of serial sections. The pattern of EGFP expression is almost identical to that of NADH-TR staining. Scale bar represents 100 µm.

**Figure 4:**
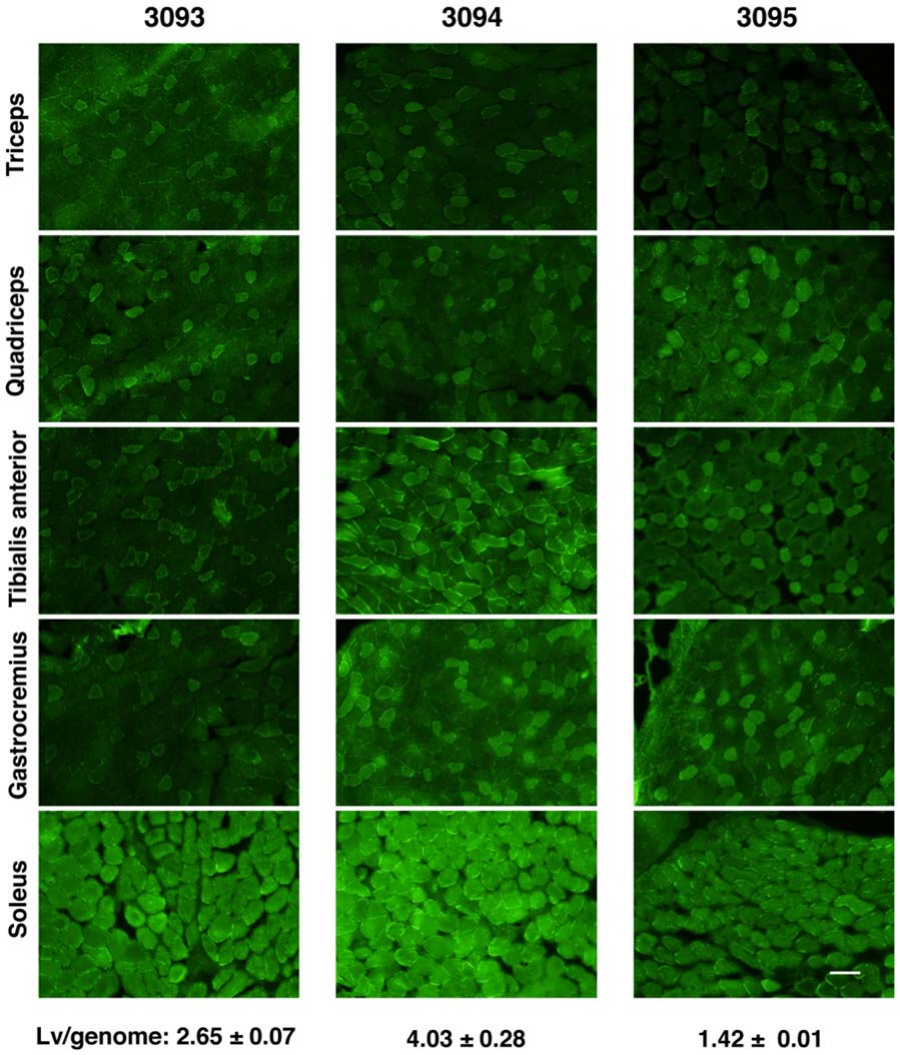
EGFP expression regulated by MSCV promoter in skeletal muscles following embryo transduction. Brachio-triceps, quadriceps, tibialis anterior, gastrocnemius, and soleus muscles of 3 different mice (3093, 3094, and 3095) from independent embryo-transductions were cryosectioned and observed under a fluorescence microscope. The average Lv-copy numbers (mean ± SEM) are shown at the bottom. Each section shows the expression of EGFP. Although the overall levels of EGFP expression varied among the three mice (transduction at the two-cell embryo stage), they each displayed a similar fiber-type expression pattern. Scale bar represents 100 µm.

To analyze the EGFP expression levels in skeletal muscles, 10-µm-thick frozen sections of the brachio-triceps, quadriceps, tibialis anterior, gastrocnemius, and solius muscles from LvMSCV-EGFP transgenic and wild type mice were observed under a fluorescence microscope (Figure 3). The fiber types were determined by NADH-TR staining, ATPase staining at pHs 10.8, 4.5, and 4.2 (Figure 3), and also confirmed with fiber type specific antibodies (Method S1, Figure S1). Various EGFP expression levels were seen at 488 nm. In the brachio-triceps, quadriceps, tibialis anterior, and gastrocnemius muscles (fast twitch), which consist mainly of type IIB and IID/X, there were EGFP expression, however a mosaic pattern in the level of expression was observed: type IID/X fibers showed higher expression than the type IIB fibers. In the soleus muscles (slow twitch), which predominantly consist of type I and type IIA fibers, almost all of the fibers strongly expressed EGFP as well as NADH-TR. In both of the tibialis anterior and the soleus muscle, the pattern of EGFP expression was almost identical to that of the NADH-TR staining. Although there were still minor variations in EGFP expression levels among the various mice due to embryo transduction variation, all of the collected muscles presented similar EGFP expression patterns (Figure 4).

**Figure 5:**
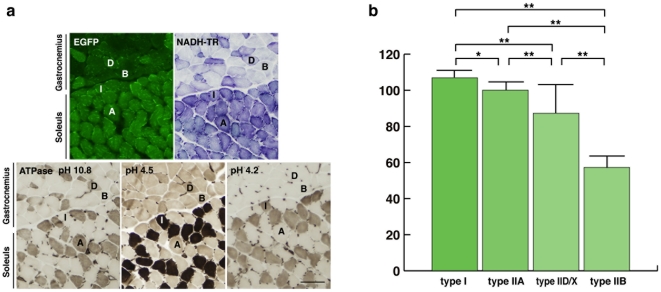
MSCV-EGFP transgenic mice showed fiber type-dependent variations in EGFP protein expression. (**a**) The expression of EGFP at the border of the gastrocnemius and soleus muscles, where all 4 fiber types, type I, type IIA, type IIB, and type IID/X (labeled I, A, B, and D, respectively), are present. Each fiber type was confirmed by NADH-TR and ATPase (at pHs 10.8, 4.5, and 4.2) staining. Bar represents 100 µm. (**b**) Bar graph showing the mean (+SEM) intensity levels of EGFP protein in each fiber type. Type I fibers and IIA fibers presented the strongest signals, type IID/X fibers had intermediate expression levels, and type IIB fibers had the weakest. Except between type I and type IIA (*p = 0.03), there were significant differences among the various fiber types (**p<0.005).

These four different fiber types can be captured together in an axial transverse section of the border of the gastrocnemius and soleus muscles, making it easy to compare the EGFP expression levels among them in the same section (Figure 5a). Quantitative analysis of fluorescence intensity showed that the strongest signals were in the type I (106.92±1.25) and IIA (100.09±1.62) fibers, intermediate signals were in type IID/X fibers (87.30±3.60), and the weakest signals were in type IIB fibers (57.29±1.71). There were significant differences among these three groups (p<0.005, Figure 5b).

## Discussion

**Figure 6:**
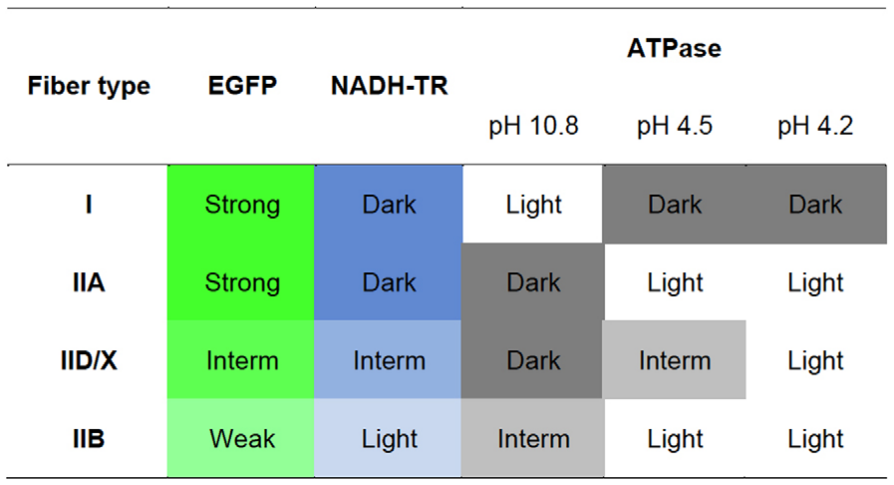
Summary of the fiber type-dependent EGFP expression in LvMSCV-EGFP mediated transgenic mice. Levels of EGFP expression and typical staining pattern of each muscle fiber type are summarized. The EGFP expression pattern was mostly identical to that of the NADH-TR staining. Fiber types were determined by the ATPase stainings with different pHs (pH 10.8, 4.5, and 4.2). Colors reflect the staining colors on muscle sections.

In our examination of the usefulness of the MSCV promoter to drive the expression of therapeutic genes, such as *dystrophin*, in skeletal muscles [6], we focused on its activity in skeletal muscle tissue and myotubes. In young mice given intramuscular injections of LvMSCV-EGFP vector into the TA muscles, type IID/X fibers expressed higher levels of EGFP than did type IIB fibers. Then, we tested this MSCV-EGFP expression cassette in transgenic mice using Lentiviral-mediated gene transduction into two-cell-stage embryos. In the transgenic mice, EGFP expression was highest in type I and type IIA muscle fibers; showed medium expression in type IID/X fibers, and was lowest in type IIB fibers (summarized in Figure 6.) When we observed the border area of the soleus and gastrocnemius muscles, where all 4 fiber types are present in one section, expression in type IID/X fibers was a little weaker than in the type I and type IIA fibers. Together, these observations demonstrate that the activity of the MSCV promoter exhibits fiber type predominance. These differences in promoter activity might arise from the type of myosin (fast or slow), amount of myoglobin, the degree of oxidative phosphorylation that the fiber undergoes, or the reliance on the glycolytic enzymes of oxidative metabolism to generate ATP.

Most murine skeletal muscles consist of mainly type IIB and type IID/X fibers, although the soleus and diaphragm consist of mainly type I and IIA muscle fibers [32], [33]. In the *mdx* mouse, tongue, heart, diaphragm, and soleus muscles exhibit the most severe dystrophic changes, such as regeneration and degeneration with fibrosis [34], [35]. The diaphragm and soleus are among the slowest of muscles in the mouse [36]. These observations suggest that the susceptibility for dystrophin-related fiber damage may be related to the fiber type predominance of each muscle, which may, in turn, be due to their glycogen levels, the ratio of skeletal myosin isoforms, or their frequency of movement. The diaphragm is also one of the most severely affected muscles in DMD patients, although some reports have suggested that mixed fiber type muscles show more necrosis and subsequent regenerations of fast fibers in biopsies from Psoas and Vastus lateralis muscles [37] or type IIB fibers were affected in early stage of DMD boys [38]. As nNOS is predominantly expressed in the type IIB fibers [39], the loss of nNOS activity caused by the dystrophin deficiency is considered to play an important role in the pathogenesis of muscle degeneration in DMD, although there is discrepancy (difference) of nNOS localization in muscle fiber types between human and rodent skeletal muscles.

The mosaic expression patterns often observed with marker genes (e.g., GFP or LacZ) may be due to the fiber type-specific promoter activities of CMV, CAG, and CK6 promoters [26], as was often shown in muscles following viral vector-based transduction. This activity variance is not negligible from the standpoint of their applications. We have shown that the MSCV promoter activity was stronger in type I and type IIA fibers found mainly in slow twitch muscles. In other words, this promoter has a high predilection for muscles that are susceptible to dystrophic damage. Thus, as this promoter achieved long-term dystrophin expression in the *mdx* mouse [6], it might be a candidate promoter for gene therapy of murine muscular dystrophy models. On the other hand, we consider that any fiber types without dystrophin expression need to be treated by dystrophin gene replacement. At the very least, additional analyses should be carried out to provide further details about the ‘ideal expression cassette’ for using it in the gene therapy of muscular dystrophies, particularly in terms of combining it with other muscle-specific promoters. A number of studies have compared different versions of promoters/enhancers derived from the muscle creatine kinase gene (MCK), such as CK6 [26] and CK7 [27]. Interestingly, those promoters showed preferential activity in fast fiber types, rather than in the slow fibers that we observed here with MSCV.

Regarding the safety issue of gene therapy using vector cassettes, muscle tissue-specific promoters are considered to be advantageous for eliminating transgene expression in other types of cells, especially antigen presenting cells [40], [41], [42], [43]. On the other hand, we propose the idea of “the right promoter for the right muscle”. At this time there may be strengths and weaknesses in all known promoters, which should be investigated and clarified. From a clinical point of view, as the weakness of respiratory muscles, including especially the diaphragm, are directly linked to patient lifespan [44], they are important targets of therapeutic approaches for DMD patients [45]. If the new candidate promoter is also activated better in the diaphragm, it may be applicable for gene or cell therapies targeting this muscle; although, an entire, body-wide therapeutic gene expression might be the best choice for DMD patients, if we could achieve it without safety problems.

As we have shown in this study, and also in a previous report [31], Lv-mediated transgenic mice were efficiently generated by infecting fertilized eggs with vesicular stomatitis virus envelope protein (VSV-G)-pseudotyped lentiviral vectors. As shown in Figure 4, there were still variable expression levels of the transgene among the lines, probably due to the integrated proviral copy number or variety of integration sites. Still, we have highlighted the usefulness of promoter analysis using these transgenic mouse lines. Although multiple proviral insertions make it difficult to establish pure-breeding transgenic lines, this might be an advantage for evaluating the nature of lentiviral vectors *in vivo*, as the situation is more similar to that of clinical gene therapy. Moreover, myofibers themselves may equalize heterogeneity of copy numbers and insertion sites of provirus, because each myofiber contains abundant myonuclei from each myoblast fusion.

In conclusion, we have shown that MSCV promoter activity in skeletal muscle is fiber-type-dependent when delivered directly by lentiviral infection as well as in transgenic mice generated by lentiviral infection. Further analysis of this promoter may determine the necessary components needed to direct the slow muscle-dominant gene expression and that are capable of coordinating with other muscle-specific expression cassettes for therapeutic application. Moreover, the Lv-mediated transgenic mouse may prove to be a useful tool for assessing the enhancer/promoter activities of a variety of different regulatory cassettes.

## Materials and Methods

### Cell cultures

293D cells (human embryonic kidney cells, kindly provided by Dusty Miller, Fred Hutchinson Cancer Research Center), NIH3T3 cells, and C2C12 cells (both provided by RIKEN BRC CELL BANK, Tukuba, Japan) were used. All cells were grown in Dulbecco's modified Eagle's medium (DMEM; Invitrogen, Carlsbad, CA) with 10% fetal bovine serum (HyClone, Logan, Utah, USA), 50 U/ml penicillin, and 50 U/ml streptomycin (Sigma, St. Louis, MO, USA). The cultures were incubated at 37°C in a humidified atmosphere containing 5% CO_2_.

### Construction of lentiviral transfer vectors

Lentiviral vectors were constructed as described previously [6]. Transfer vectors were constructed by inserting the corresponding coding sequences into the polylinker of the pRRL-cPPT-CMV-X-PRE-SIN vector [46]. For a transfer vector, the human CMV promoter was replaced with the MSCV promoter [47].

### Lentiviral vector preparation and titration

Lentiviral vectors were generated as described previously [1], [6]. VSV-G-pseudotyped lentiviral vectors were produced by co-transfecting the appropriate plasmids into 293D cells, and purified by ultracentrifugation as described [6], [9], [10], [48]. The titer of viruses containing the reporter gene was measured by transducing 293D and NIH3T3 cells with serial dilutions of vector preparations, and also by quantitative RT-PCR methods [21], which were adjusted with flow cytometry (FACS) data of a well-known titered control EGFP vector (LvMSCV-EGFP) as previously described [6], [9]. Briefly, 2×10^5^ cells were transduced with 0.05 µl of LvMSCV-EGFP vector and cultured in fresh DMEM plus 10% fetal calf serum (FCS, HyClone, Logan, Utah, USA) for 2 days before FACS analysis in triplicate. 50% of the cells expressed EGFP in the cultures. The titer of these vector stocks was estimated by measuring viral p24 gag antigen using the HIV-1 p24 Antigen ELISA Assay kit (ZeptoMetrix Corporation Inc., Buffalo, NY, USA).

### Mouse strains

C57BL/10, C57BL/10 *mdx* (Central Institute for Experimental Animals, Kawasaki, Japan) and B6D2F1 (*Research Institute for Microbial Diseases, Osaka University, Osaka, Japan*) strains of mice were used. All animal experiments were approved by the Kumamoto University Committee on Animal Research (permit number C22-176) and the Osaka University Committee on Animal Research (permit number 2589). The mice were housed in the Center for Animal Resources and Development (CARD) of Kumamoto University in rooms that were maintained at 22±2°C, 50±10% relative humidity, and a 12/12 h light/dark cycle. They had free access to drinking water and standard chow, and were sacrificed at 3 months.

### Intramuscular injection with LvMSCV-EGFP

Vectors were injected into muscles as described previously [48]. Briefly, 2-day-old mice were put on ice to anesthetize with hypothermia, and then injected with 5 µl of viral preparations with titers of 1.0×10^9^ TU/ml into the right and left TA muscles. Four weeks after intra-muscular administration, the TA muscles were harvested; some were cryosectioned for histology, and some were used for primary cultures.

### Histological staining with H&E, NADH-TR, PAS, and ATPase staining

Routine H&E, NADH-TR, and ATPase staining were performed as described previously [30]. Briefly, the various fiber types were determined by NADH-TR staining and differential ATPase staining at different preincubation pHs. ATPase at pH 10.8 distinguishes type I (light staining) from type II (dark staining) fibers; pH 4.5 distinguishes type I (dark staining) from IIA (light staining) and IIB (medium staining) fibers; and pH 4.2 distinguishes IID/X (medium staining) from IIA or IIB (light staining) fibers. The three preincubation reagents were (1) 20 mM sodium barbital, 36 mM CaCI_2_, pH 10.8, (2) 50 mM sodium acetate, 30 mM sodium barbital brought to pH 4.5 with HCI, and (3) the same as (2) but adjusted to pH 4.2. The sections were preincubated for 15 min at pH 10.8 and 5 min at the acid pHs. After preincubation, the sections were incubated for 45 min in 20 mM sodium barbital, pH 9.5, containing 9 mM CaCl_2_ and 2.7 mM ATP; rinsed in 2 changes of 1% CaCl_2_ (1 min each); immersed for 2 min in 2% CaCl_2_; and rinsed with several changes of tap water. After staining with 1% (NH_4_)_2_S, the sections were washed with several changes of tap water, dehydrated with ethanol, cleared in xylene, and mounted in balsam.

### Isolation and culturing of muscle mononuclear cells

Mononuclear cells were harvested from the TA hindlimb muscles of LvMSCV-EGFP-injected mice at 2 mo, as well as from the TA muscles of age-matched, wild type mice as a control for culturing as described previously [6]. Briefly, TA muscles were excised, minced, and digested in phosphate buffered saline (pH 7.2 GIBCO, Invitrogen, Grand Island, NY) with final concentrations of 1 mM CaCl_2_ and 0.2% collagenase II (Worthington Biochemical Corporation, Lakewood, NJ) at 37°C for 45 min, and then filtered through 70- and 40-µm nylon filters (BD Falcon, Franklin Lakes, NJ). Mononuclear cells were cultured on 0.67% gelatin-coated plates in medium containing F10 medium (GIBCO, Invitrogen, Grand Island, NY) with 10 mM CaCl_2_ (F10C) plus 15% horse serum (GIBCO, Invitrogen, Grand Island, NY) and 5 ng/ml recombinant human basic FGF-2 (R&D systems, Minneapolis, MN). At day 7, differentiation was induced by rinsing the cultures and switching them to medium containing F10C with 1.5% horse serum and 6 mg/ml insulin for 48 h, followed by re-feeding with F10C with 15% horse serum and insulin [6], [49]. At day 14, cells were observed under a fluorescence microscope (DP70-WPCXP, Olympus, Tokyo, Japan).

### Treatment of embryos and generation of transgenic mice

Lentiviral transduction in two-cell-stage embryos was described previously [31]. Briefly, B6D2F1 females were superovulated by intraperitoneal injection of pregnant mare's serum gonadotropin (5 U) and 48 h later with human chorionic gonadotropin (5 U), and then mated with B6D2F1 males. Two-cell-stage embryos were collected from the oviducts of the copulated females 36 h after injection of human chorionic gonadotropin. To remove the zona pellucida, the embryos were placed in acidic Tyrode's solution [50] for 30 s to 1 min. After dissociation of the zona pellucidae was confirmed, embryos were washed three times with kSOM [51] and incubated at 37°C for 2.5 days in a 5-µl drop of kSOM containing viral vectors at multiplicity of infection of 10^5^. Blastocysts developed from infected embryos were transferred into 2.5 day pseudo-pregnant females [31]. The pups were delivered via Caesarean section at pregnant day 20 (Table S1).

### Detection of proviral DNA copies with PCR and Q-PCR

DNA was isolated using the DNeasy Blood & TissueFlexiGene DNA kit (QIAGEN Science, Maryland, MD) from the tail tips of weaned pups, and subjected to 40 rounds of PCR amplification with primers EGFP for (5′-GCCACCATGGTGAGCAAGGGCGAG-3′) and EGFP rev (5′-TCACCTTGATGCCGTTCTTCT-3′) to check the existence of the EGFP transgenes [31].

The number of proviruses per genome in transgenic lines was determined by the Q-PCR method as described previously [21]. Briefly, diluted DNA was analyzed by real time PCR using probe PRE (5-FAM AGCTCTCTCGACGCAGGACTCGGC-TAMRA-3) and primers F-PRE (5- ACCTGAAAGCGAAAGGGAAAC-3), R-PRE (5-CACCCATCTCTCTCCTTCTAGCC-3) [52]. The genome copies in each sample were determined using probe LDLR (5-FAM-TGCCAGGATGGCAAGTGCATCTCC-TAMRA-3) and primers F-LDLR (5-CGTGCTCCCAGGATGACTTC-3), and R-LDLR (5-CTCCATCACACACAAACTGCG-3), all obtained from Applied Biosystems (Foster City, CA). The standard curve was generated using L-mouse-LDLR (low-density lipoprotein receptor) plasmid DNA carrying the LDLR gene sequence between +9 to +69 inserted into pRRL-cPPT-X-PRE-SIN [46]. The number of proviruses per genome in each sample was calculated using the formula: copies of proviruses/copies of LDLR/2. Each transgenic line was analyzed in triplicate.

### Immunostaining of frozen muscle sections

Air-dried cryosections were rinsed with PBS and blocked with 2% normal goat serum. Sections were then incubated with rabbit anti-EGFP antibody (1∶1000; Molecular Probes, Eugene, OR), and then with the secondary, Alexa488-anti-rabbit antibody (1∶1200; Molecular Probes). 4′, 6-diamidino-2-phenylindole, dihydrochloride was used for fluorescent nuclear staining (DAPI, 500 ng/ml; Molecular Probes) and the sections were mounted with VECTASHIELD (Vector lab, Burlingame, CA). Stained sections were observed using an optical fluorescence microscope (DP70-WPCXP, Olympus, Tokyo, Japan).

### Images and statistical analysis

Images of stained muscle sections were analyzed using Image J (Version 1.43r, Wayne Rasband, National Institutes of Health). The EGFP signals in each fiber type (Type I, IIA, IIB, IID/X) were measured, and then averages (means ± SEM) of signal intensities in each fiber type were compared. Statistical analysis was performed using a commercially available software package (JMP, Version 8.0.2, SAS Institute Inc., USA). *T*-tests were used to detect differences between groups. *P*- values <0.005 indicated statistically significant differences.

## Supporting Information

Figure S1 **LvMSCV-EGFP transgenic mouse muscle showed fiber type-dependent variations in EGFP protein expression.** The serial sections of a soleus muscle from LvMSCV-EGFP transgenic mouse expressed EGFP strongly in both type I and type IIA (labeled I, A, respectively) fibers, confirmed by immunofluorescent staining for myosin heavy chain type I and type IIa, NADH-TR staining, and ATPase staining (at pHs 10.8, 4.5, and 4.2). Bar represents 100 µm.
(TIF)

Table S1 **Efficiency of embryo transduction with lentiviral vectors.** The efficiency of generating Lv-mediated transgenic mice is shown. The zona pellucida was removed from two-cell-stage embryos of B6D2F1 mice, and they were transduced with LvMSCV-EGFP at a multiplicity of infection of 10^5^, then transferred into 2.5-day pseudo-pregnant females. The existence of transgenes was determined by PCR.
(DOC)

Methods S1 **Immunofluorescent staining for fiber type specific myosin heavy chains.**
(DOC)
