# Supplementary material for: Muscle Fiber Type-Predominant Promoter Activity in Lentiviral-Mediated Transgenic Mouse
Source: PLoS One. 2011 Mar 18;6(3):e16908. doi: 10.1371/journal.pone.0016908 (PMC3060803; doi:10.1371/journal.pone.0016908)
Supplement: Table S1 — Efficiency of embryo transduction with lentiviral vectors. The efficiency of generating Lv-mediated transgenic mice is shown. The zona pellucida was removed from two-cell-stage embryos of B6D2F1 mice, and they were transduced with LvMSCV-EGFP at a multiplicity of infection of 105, then transferred into 2.5-day pseudo-pregnant females. The existence of transgenes was determined by PCR. (DOC) [file pone.0016908.s002.doc]

Table S1. Efficiency of embryo transduction with lentiviral vectors

| *Number of mothers* | *Transduced lentiviral vector* | *No. of eggs*  *transplanted* | *No. of pups delivered* | *No. of positive transgenes* |
| --- | --- | --- | --- | --- |
| 3 | *LvMSCV-EGFP* | 60 | 17 | 8 (♂4 ♀4) |
