# Supplementary material for: Muscle Fiber Type-Predominant Promoter Activity in Lentiviral-Mediated Transgenic Mouse
Source: PLoS One. 2011 Mar 18;6(3):e16908. doi: 10.1371/journal.pone.0016908 (PMC3060803; doi:10.1371/journal.pone.0016908)
Supplement: Methods S1 — Immunofluorescent staining for fiber type specific myosin heavy chains. (DOC) [file pone.0016908.s003.doc]

Methods S1

*Immunofluorescent staining for fiber type specific myosin heavy chains.*

Immunofluorescent staining for type I and type IIA myosin heavy chain (MHC) was performed as described previously [1]. Briefly, frozen muscle sections were fixed in methanol and blocked with M.O.M. blocking kit (Vector lab, Burlingame, CA), then incubated with BA-D5 antibody for type I MHC (1:100, Developmental Studies Hybridoma Bank (DSHB), Iowa-city, Iowa, USA) followed by Alexa Fluor 594 goat anti-mouse IgG2b (1:1000, Molecular Probes, Carlsbad, CA, USA) or with SC-71 antibody for type IIA MHC (1:100, DSHB) followed by Alexa Fluor 594 goat anti-mouse IgG1 (1:1000, Molecular Probes).

1. Mendler L, Pinter S, Kiricsi M, Baka Z, Dux L (2008) Regeneration of reinnervated rat soleus muscle is accompanied by fiber transition toward a faster phenotype. J Histochem Cytochem 56: 111-123.
